# Short-Term Consumption of Low-Molecular Weight Polyphenols (Oligonol) Is Associated with Improved Post-Exercise Recovery in Healthy Young Men: A Randomized Single-Blind Crossover Study

**DOI:** 10.3390/antiox15020250

**Published:** 2026-02-14

**Authors:** Hyojin Kim, Jihyun Park, Su Min Hwang, Sumin Oh, Byounghyeon Kim, Jin-Hee Woo, Oh Yoen Kim

**Affiliations:** 1 Department of Health Sciences, Graduate School of Dong-A University, Sahagu, Nakdongdaero 550 beon-gil, Busan 49315, Republic of Korea; 2271961@donga.ac.kr (H.K.); 2277669@donga.ac.kr (J.P.); 2272071@donga.ac.kr (S.M.H.); 832955@donga.ac.kr (S.O.); 2 Department of Physical Education, College of Arts and Physical Education, Dong-A University, Sahagu, Nakdongdaero 550 beon-gil, Busan 49315, Republic of Korea; qudgus20@dau.ac.kr; 3 Department of Food Science and Nutrition, Dong-A University, Sahagu, Nakdongdaero 550 beon-gil, Busan 49315, Republic of Korea

**Keywords:** low-molecular weight polyphenol, exercise load, fatigue, lactate, oxidative stress, malondialdehyde

## Abstract

Polyphenols have been suggested to aid exercise recovery through antioxidant properties, but their efficacy remains controversial, partly due to limited bioavailability. This study investigated whether low-molecular weight polyphenols (LMWPs, oligonol) influence metabolic responses related to fatigue and oxidative stress during and after a maximal exercise test in healthy young men. A randomized, single-blind crossover design includes a placebo, a single dose of LMWP (S-LMWP), and 5-day LMWP (5-LMWP) intervention with ≥2-week washouts. Ten eligible participants completed all conditions. Exercise performance, fatigue-related metabolic parameters, and oxidative stress markers were measured before, immediately after, and 30 min after exercise. Heart rate and lactate were additionally assessed for 5 min post-exercise. Exercise performance and anthropometrics did not differ among conditions. However, both LMWP groups showed significantly lower blood lactate at 30 min recovery compared with the placebo group (placebo: 17.09 ± 1.29; S-LMWP: 8.36 ± 0.73; 5-LMWP: 9.18 ± 0.60; *p* = 0.005). Malondialdehyde (MDA), elevated immediately post-exercise, returned closer to baseline at 30 min in the LMWP groups, particularly S-LMWP. Percent MDA change was significantly lower in the S-LMWP group than in the placebo group (placebo: 26.80 ± 3.01; S-LMWP: −8.41 ± 4.86; *p* = 0.007). Short-term LMWP supplementation did not affect performance or immediate responses but was associated with a more favorable recovery profile, including lower lactate and faster normalization of oxidative stress markers. Larger controlled studies are needed to confirm these findings.

## 1. Introduction

Regular and moderate exercise has beneficial effects on health by reducing the risk of cardiovascular disease, cancer, osteoporosis, and diabetes [1,2]. In particular, it has an anti-stress effect by producing intermittently low or intermediate levels of reactive oxygen species (ROS) and regulating the signaling pathways at cellular levels [3]. However, acute and intense aerobic or anaerobic exercise can produce high levels of ROS which can induce oxidative stress, thereby contributing to the wasting of antioxidants in the body and affecting health conditions such as recovery rate from fatigue or damage, etc. [4]. Oxidative stress is caused by an imbalance between the production of ROS and the capacity to detoxify reactive intermediates or the antioxidant performance, and oxidative stress resulting from the overproduction of ROS can be a crucial mediator of damage to cellular components including lipids, membranes, proteins, and DNA [5]. Also, ROS produced during exercise contributes to inflammation-related muscle damage and delayed impairment of skeletal muscle function [6]. Accumulation of lactate during intense exercise is associated with a decrease in intramuscular pH due to hydrogen ion generation, which can disturb acid–base equilibrium and impair muscle contractile function [7]. It has been reported that efficient clearance of fatigue-related metabolites, such as lactate and hydrogen ions, can accelerate recovery and improve exercise performance [8,9].

Polyphenols are antioxidants abundant in plants and have potential to be beneficial for health [10,11,12]. They are well-known for their working as radical scavengers and metallic chelators, but various factors in the body, such as food matrix interacting therein, liver capacity for metabolism, and gut microbiota, can result in low bioavailability [13,14]. Nevertheless, both acute and chronic polyphenol supplementation can improve exercise capacity and performance [14,15,16]. This is thought to be associated with the recovery from exercise-induced muscle inflammation and oxidative stress [14]. Cases et al. reported that supplementation with PerfLoad, an extract from grape (*Vitis vinifera* L.), pomegra/nate (*Punica granatum* L.), and green tea (*Camellia sinensis* L. Kuntze), at 60 min before exercise increased peak power output, average power output, and the activities of catalase in blood and plasma superoxide dismutase without inducing fatigue or increasing heart rate [15]. According to Murphy CA et al., 7 days of blackcurrant intake improved overall performance capacities in repetitive short-distance cycling without changes in blood lactate concentration and heart rate [16]. However, despite extensive research on antioxidant and anti-inflammatory dietary supplements in exercise science, their efficacy in attenuating exercise-induced fatigue and oxidative stress remains controversial [17,18,19,20]. Conventional antioxidants, such as vitamins C and E, CoQ10, and omega-3 fatty acids, have shown limited or inconsistent benefits, and several studies have reported that chronic supplementation may blunt exercise-induced oxidative signaling and impair physiological adaptations, particularly in trained or elite athletes [18,21,22].

Polyphenols are beneficial for increasing exercise performance with their antioxidant capacity, but they still have low bioavailability in the body, which needs to be improved [13,14,15,16]. To address the limited bioavailability of polyphenols, strategies to reduce the molecular size of high-molecular weight (HMW) polyphenols have been developed to enhance intestinal absorption, supported by recent evidence highlighting the biological potential of oligomeric proanthocyanidins and earlier studies demonstrating the hydrogenolytic depolymerization of polymeric proanthocyanidins into low-molecular weight forms, with oligonol as one such example [23]. Oligonol is an oligomerized polyphenol produced through the controlled oligomerization of proanthocyanidins (PCs), and it contains catechin-type monomers as well as oligomeric PCs [23,24]. Because PCs are polymers of catechin, their intestinal absorption is limited due to their high molecular weight when orally consumed [23]. Therefore, oligonol, a low-molecular weight polyphenol derived from lychee fruit, was developed by converting HMW PC into low-molecular weight forms [25]. Oligonol also showed significantly higher blood antioxidant capacity than (+)-catechin and (−)-epigallocatechin 3-O-gallate in normal rats after one week of administration at a dose of 20 mg/kg [26]. Furthermore, oligonol, as an antioxidant, has been shown to ameliorate various oxidative stress-induced diseases by suppressing inflammatory cytokines and related signaling proteins [27,28,29,30]. In exercise-induced oxidative stress, oligonol intake significantly decreased interleukin (IL)-6, IL-1β, and cortisol induced by 60 min of running at 75% of VO_2_max intensity in men, and also increased high-intensity interval exercise performance [31,32]. These results suggest that the antioxidant capacity of oligonol may help maintain exercise performance during high-intensity training [32]. In other words, low-molecular weight polyphenols (LMWPs) represent a distinct class of antioxidant compounds with improved bioavailability and rapid absorption compared with conventional polyphenols [23,24,25]. Oligonol, a standardized LMWP formulation, has been shown to exert antioxidant and anti-inflammatory effects without completely abolishing reactive oxygen species (ROS) signaling [26,27,28,29,30,31,32]. These properties suggest that LMWPs may be more suitable for short-term or acute interventions aimed at attenuating excessive oxidative stress and fatigue responses during recovery from high-intensity exercise.

As mentioned above, interest in the effects of LMWP on fatigue-related substances and oxidative stress is increasing. However, limited information is available regarding the acute and short-term effects of LMWP supplementation on fatigue-related metabolic parameters and oxidative stress responses during maximal exercise in healthy individuals. Therefore, this study investigated whether single or short-term LMWP consumption affects exercise-induced fatigue and oxidative stress responses during a maximal exercise test using a randomized, blinded crossover design.

## 2. Materials and Methods

### 2.1. Study Subjects

Healthy males in their twenties were recruited through advertisements. Among the 20 volunteers, individuals with recent injuries, diagnosed chronic diseases, or who were unable to perform an exercise load test were excluded. Finally, 10 males were enrolled in the study. The minimum sample size for this study was calculated, with an effect size of f = 0.40, α value = 0.05, and power (1-β) = 0.8 level, using the G*Power 3.1.9.4 software [33]. All participants signed consent forms and received research details, including the study objectives, procedures, benefits, potential risks, data use and storage, and data disposal instructions. This study was approved by the Institutional Review Board of Dong-A University (2-1040709-AB-N-01-202212-HR-052-04).

### 2.2. Study Design

This study employed a randomized single (participants)-blind crossover design with a 2-week washout period between trials. During the placebo phase, all participants received 500 mL of water provided in opaque bottles. In both the single-dose LMWP phase (S-LMWP) and the 5-day LMWP phase (5-LMWP), participants received opaque bottles containing 500 mL of water with 200 mg of oligonol, a polyphenol polymer derived from the oligomerization of lychee fruit pericarp (Amino Up Chemical Company, Sapporo, Hokkaido, Japan). All beverages were flavored with strawberry essence to ensure blinding and prevent participants from distinguishing between drinks. No other nutrients or energy were included in any of the beverages. Therefore, the placebo and experimental drinks were nutritionally identical, with the only difference being the presence of oligonol in the S-LMWP and 5-LMWP phases. In the placebo and S-LMWP phase, participants consumed the beverage prior to the exercise load test on the day of their visit. In the 5-LMWP phase, participants consumed the beverage daily for five consecutive days before the visit and did not consume it on the day of the test. Study participants were instructed to maintain their usual diet and lifestyle habits during the intervention period in order to preserve consistent experimental conditions. Intensive physical activity and alcohol consumption, which could potentially affect exercise testing and study outcomes, were prohibited throughout the experimental period. Participants were also instructed to refrain from consuming any food or beverages other than water after 9:00 p.m. the day before the test until the morning of the test day. Before the test, participants completed the general characteristics and physical activity readiness questionnaire.

### 2.3. Nutrition Quotient

Nutrition quotient (NQ) is an index used to assess the eating behavior, diet quality, and nutritional status of individuals or groups. In this study, the adult version of the NQ survey, developed and validated by the Korean Nutrition Society, was used to evaluate participants’ usual dietary habits prior to the test [34,35]. The questionnaire consists of three domains—balance, moderation, and practice—from which the overall NQ score is derived. Based on the established scoring criteria, participants were categorized into three levels: high (75–100%), middle (25.0–74.9%), and low (0–24.9%).

### 2.4. Exercise Load Test

An exercise load test was performed after body composition measurement. The VO_2_max was measured using Quark CPET gas analysis (COSMED, Rome, Italy) during a test on the treadmill (T150DE, COSMED, Werneck, Germany). VO_2_max is defined when meeting at least two of the following criteria: (1) The subject can no longer continue the exercise or increase exercise intensity, (2) heart rate or oxygen consumption does not significantly increase anymore, (3) a respiratory exchange ratio (RER) is more than 1.10, or (4) a rating of perceived exertion (RPE) is more than 17. All participants put on a face mask during the test and underwent a maximal exercise load test using the Bruce treadmill protocol. During the test, environmental factors such as temperature and humidity were recorded to ensure that all trials were conducted under the same conditions, as far as possible. Grip strength was measured by pulling up to maximum for 2–3 s, repeating the process twice for each hand, and recording the highest value in 0.1 kg units using an electronic grip force meter (TAKEI, Tokyo, Japan). Back muscle strength was measured by lifting the handle of the Hellmas II digital back dynamometer (O2RUN Co., Ltd., Seoul, Republic of Korea) to maximum for about 3 s, repeating this process twice, and recording the highest value in 0.1 kg units.

### 2.5. Anthropometric Parameters and Blood Pressure

All the participants were asked to follow the specific guidelines before the measurement: neither intensive physical activity nor alcohol consumption within 12 h, keeping the fasting status for at least 12 h, and urination within 30 min before the measurement. All participants were measured for their body composition (body fat mass (BFM), body fat percentage (BFP), body mass index (BMI), body weight, height, lean body mass (LBM), skeletal muscle mass (SMM)) using an impedance body fat analyzer (Accuniq BC720, SELVAS Healthcare, Seoul, Republic of Korea) before, after, and at 30 min of rest after a maximal exercise test. Blood pressure was measured in the upper arm using an automatic electronic blood pressure monitor (Automatic Blood Pressure Monitor-HEM7121, OMRON, Kyoto, Japan) at rest. The heart rate was also measured using HERAFit PRO (Health-One Co., Ltd., Goyang, Republic of Korea) before and after exercise, and at 1, 2, 3, 4, 5, and 30 min of rest, at the upper arm.

### 2.6. The Measurement of Lactate and Glucose in Whole Blood

Blood lactate levels were simply measured using a blood lactate analyzer (Lactate PRO2-LT1730, Arkray, Kyoto, Japan). A total of 5 μL of whole blood was collected from the fingertip before and after exercise, and at 1, 2, 3, 4, 5, and 30 min of rest. Blood glucose levels were measured using a blood glucose test strip (BAROZEN2-GM01IAC, Handok Inc., Seoul, Republic of Korea) before a maximal exercise test.

### 2.7. Blood Collection

Blood samples were collected from the median cubital vein into vacutainers by trained nurses for the analysis of biochemical markers before, after, and at 30 min of rest after a maximal exercise test. The samples were centrifuged at 3000 rpm at room temperature for 15 min, and then the separated serum was stored in a −80 °C deep freezer until the analysis.

### 2.8. Fatigue Parameters

Uric Acid (UA) was measured by the enzymatic method, and phosphorus, lactate dehydrogenase (LDH), and creatine kinase (CK) were measured using a UV assay with a LABOSPECT 008AS (Hitachi High-Tech Co., Ltd., Tokyo, Japan).

### 2.9. Oxidative Stress-Related Markers

Malondialdehyde (MDA) concentrations in serum were measured by thiobarbituric acid reactive substances (TBARSs), which are the end products of lipid peroxidation using a kit (OxiSelect™ TBARS Assay Kit, Cell Biolabs, San Diego, CA, USA), and were analyzed using a microplate reader at 540 nm. Serum-oxidized LDL (ox-LDL) levels were measured using an Oxidized LDL ELISA kit (Mercodia, Uppsala, Sweden), and the wavelength was set to 450 nm.

### 2.10. Statistical Analysis

Statistical analysis was performed using SPSS version 27.0 (SPSS Inc., Chicago, IL, USA). The anthropometric parameters, exercise performance, and glucose level were analyzed using Friedman test (non-parametric ANOVA) for overall comparison among three phases (placebo, S-LMWP and 5-LMWP). Heart rate, blood lactate level, fatigue metabolism, and oxidative stress data were analyzed using a repeated-measures one-way analysis of variance (RM-ANOVA) for the interaction between phase (treatment) and time. We used Muchly’s test to determine the homogeneity of variance. If Muchly’s test was not the homogeneity of variance (*p* ≤ 0.05), Wilks’s lambda test was used. Otherwise, sphericity-assumed values were used. All data were analyzed using the Wilcoxon signed-rank test (non-parametric paired *t*-test) for the comparison between two phases. The relationship between oxidative stress and biochemical markers was tested using Spearman correlation analysis. Data were presented as mean ± standard error. A *p*-value < 0.05 was considered as statistically significant.

## 3. Results

### 3.1. General Information of the Study Subjects

General information including age, height, body weight, BMI, LBM, SMM, BFM, BFP, and NQ scores of subjects at baseline are presented in Table 1. Study subjects were found obese based on their mean BMI, but they were overweight based on their mean BFP. Furthermore, the average NQ values of all parts corresponded to the middle level of the categories. Table 2 presents the anthropometric parameters of participants according to LMWP consumption. Overall comparisons using the Friedman test revealed no significant differences among phases for most variables except body fat percentage (BFP) (*p* = 0.031). However, post hoc pairwise comparisons using the Wilcoxon signed-rank test showed that body weight and BMI were significantly lower in the 5-LMWP phase compared with the S-LMWP phase. Similarly, body fat mass (BFM) and BFP were significantly lower in the S-LMWP phase than in the placebo phase. No significant differences were observed in lean body mass (LBM) or skeletal muscle mass (SMM) among the three phases.

### 3.2. Exercise Performance, Fasting Glucose, and Blood Pressure of Subjects According to LMWP Consumption

Table 3 presents exercise performance, fasting glucose, and blood pressure of the participants according to LMWP consumption. Exercise performance variables included maximal oxygen consumption (VO_2_max), maximal heart rate (HRmax), exercise time (ET), anaerobic threshold time (AT), left-hand grip strength (GL), right-hand grip strength (GR), and back muscle strength (BMS). Overall comparisons revealed that HRmax differed significantly among phases (*p* = 0.006), with higher values observed in the 5-LMWP phase compared with the S-LMWP phase. Grip strength of both hands (GL and GR) was significantly lower in both LMWP phases compared with the placebo phase. Systolic blood pressure (SBP) also showed significant differences among phases, with lower values in the 5-LMWP phase compared with the placebo phase. Diastolic blood pressure (DBP) was significantly lower in both LMWP phases than in the placebo phase. In contrast, VO_2_max, ET, AT, BMS, and fasting glucose levels did not differ significantly among the three phases.

### 3.3. Comparison of Heart Rate Changes According to LMWP Consumption

Table 4 presents the time-dependent changes in heart rate according to LMWP consumption. Heart rate was measured in each phase before and immediately after exercise, and at 1, 2, 3, 4, 5, and 30 min of rest. Heart rate changed significantly over time in all phases (*p*0 < 0.001). However, there were no significant interactions between time and phases for heart rate changes (*p*1 = 0.293). Post hoc pairwise comparisons revealed that heart rate was significantly higher in the 5-LMWP phase than in the placebo phase at 1 min (*p* = 0.009) and 2 min (*p* = 0.041) of rest. After 5 min of rest, heart rate was also significantly higher in the 5-LMWP phase than in the S-LMWP phase (*p* = 0.041).

### 3.4. Comparison of Blood Lactate Levels According to LMWP Consumption

**Figure 1:**
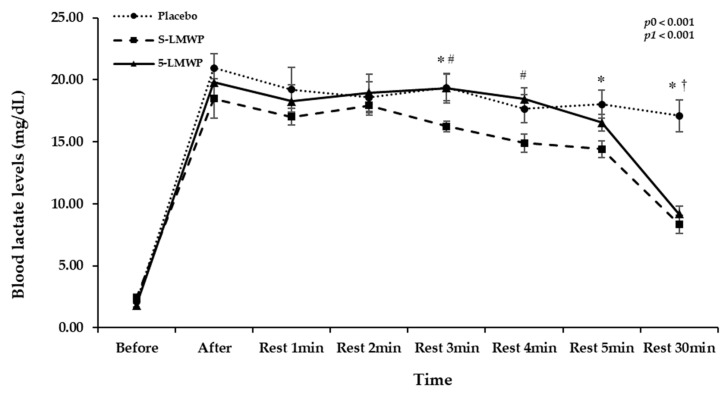
Time-dependent blood lactate levels according to LMWP consumption. Data are presented as mean ± standard error (S.E.). A *p*-value < 0.05 was considered statistically significant. The time effect (*p*0) and the time × treatment interaction effect (*p*1) were evaluated using repeated-measures ANOVA based on Wilks’s lambda. Post hoc pairwise comparisons were conducted *using* Wilcoxon signed-rank test (non-parametric paired *t*-test). * Significant difference between the placebo and S-LMWP. ^#^ Significant difference between S-LMWP and 5-LMWP. ^†^ Significant difference between the placebo and 5-LMWP. After, immediately after the maximal exercise test; Before, before the maximal exercise test; 5-LMWP, 5-day consumption of low-molecular weight polyphenol; Rest, 30 min rest after the exercise; S-LMWP, single consumption of low-molecular weight polyphenol.

Figure 1 presents blood lactate levels according to LMWP consumption before and immediately after exercise, and at 1, 2, 3, 4, 5, and 30 min of rest. Blood lactate levels in each phase were significantly changed in a time-dependent manner (*p*0 < 0.001). In addition, significant interaction between phases and time for blood lactate levels was observed (*p*1 < 0.001). Specifically, post hoc pairwise comparisons revealed that blood lactate levels were significantly lower in the S-LMWP phase compared to the placebo phase at 3, 5, and 30 min after rest (*p* < 0.05). In particular, they were significantly lower in both the S-LMWP and 5-LMWP phases compared to the placebo phase at 30 min of rest (*p* = 0.005).

### 3.5. Comparison of Fatigue Metabolism and Oxidative Stress-Related Markers Levels According to LMWP Consumption

Table 5 shows the serum levels of fatigue metabolism and oxidative stress-related markers levels according to the LMWP consumption before and immediately after exercise, and at 30 min of rest. All variables were significantly changed in a time-dependent manner (*p*0 < 0.001), but the significant interaction between treatment and time was only observed in the CK (*p*1 = 0.008). Post hoc pairwise comparisons revealed that UA levels were significantly lower in the S-LMWP phase compared to the placebo phase at 30 min of rest after exercise (*p* = 0.028). The CK levels were significantly higher in the S-LMWP phase compared to the placebo phase at all time points (*p* < 0.05). However, CK-MB and ox-LDL levels were not significantly different among the three phases. Change values of CK-MB and ox-LDL were significantly different among the three phases (*p*2 = 0.041, *p*2 = 0.006, respectively)

### 3.6. Changes of Phosphorus and LDH Levels According to LMWP Consumption

**Figure 2:**
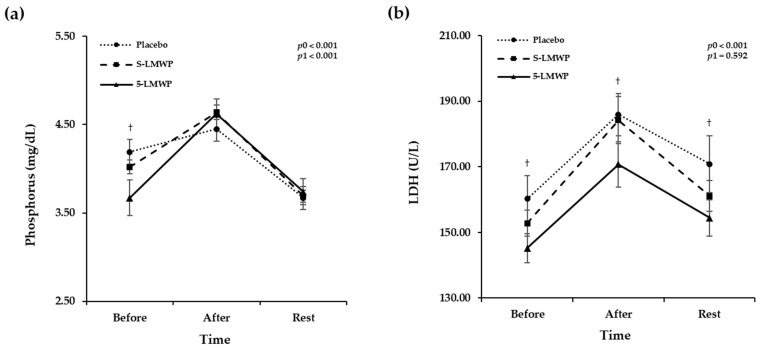
Serum levels of phosphorus and LDH according to LMWP consumption. The effects of low-molecular weight polyphenol (LMWP) consumption on serum levels of phosphorus (**a**) and LDH (**b**). Data are presented as mean ± standard error (S.E.). A *p*-value < 0.05 was considered statistically significant. The homogeneity of variance was performed using Muchly’s test. The time effect (*p*0) and the time × treatment interaction effect (*p*1) were evaluated using repeated-measures ANOVA based on Wilks’s lambda. Post hoc pairwise comparisons were conducted using Wilcoxon signed-rank test (non-parametric paired *t*-test). ^†^ Significant difference between the placebo and 5-LMWP (a, *p* = 0.036; b, *p* < 0.05). After, immediately after the maximal exercise test; Before, before a maximal exercise test; LDH, lactate dehydrogenase; 5-LMWP, 5-day consumption of low-molecular weight polyphenol; Rest, 30 min rest after the exercise; S-LMWP, single consumption of low-molecular weight polyphenol.

Figure 2 shows changes in phosphorus and LDH levels in serum according to the LMWP consumption before and immediately after exercise, and at 30 min of rest. Both phosphorus and LDH levels were significantly changed in a time-dependent manner (*p*0 < 0.001). In addition, significant treatment and time interactions were observed in serum phosphorus changes (*p*1 < 0.001), but not in LDH changes (*p*1 = 0.592). A *p*ost hoc pairwise test revealed that phosphorus levels were significantly lower in the 5-LMWP phase compared to the placebo phase before exercise (*p* = 0.036) (Figure 2a). The LDH levels were significantly lower in the 5-LMWP phase compared to the placebo phase at all time points (*p* < 0.05) (Figure 2b).

### 3.7. Changes in MDA Levels According to LMWP Consumption

**Figure 3:**
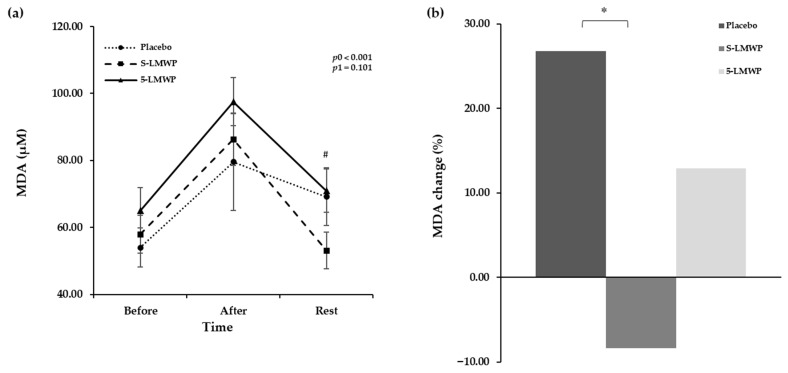
Serum levels of MDA according to LMWP consumption. The effects of low-molecular weight polyphenol (LMWP) consumption on serum levels of malondialdehyde (MDA) (**a**), and changed percentage of serum MDA levels before and at 30 min of rest after exercise (**b**). Data are presented as mean ± standard error (S.E.). A *p*-value < 0.05 was considered statistically significant. The time effect (*p*0) and the time × treatment interaction effect (*p*1) were evaluated using repeated-measures ANOVA based on Wilks’s lambda. Post hoc pairwise comparisons were conducted using Wilcoxon signed-rank test (non-parametric paired *t*-test). ^#^ Significant difference between S-LMWP and 5-LMWP (*p* = 0.022). * Significant difference between the placebo and S-LMWP (*p* = 0.007). After, immediately after a maximal exercise test; Before, before the maximal exercise test; 5-LMWP, 5-day consumption of low-molecular weight polyphenol; MDA, malondialdehyde; Rest, 30 min rest after the exercise S-LMWP, single consumption of low-molecular weight polyphenol.

Figure 3a presents the changes in serum MDA before and after exercise, and at 30 min rest after exercise, and Figure 3b shows the changed percentage of MDA levels before exercise and at 30 min rest after exercise according to LMWP consumption. The MDA levels were significantly changed in a time-dependent manner (*p*0 < 0.001), but no interaction between the treatment and time was observed (*p*1 = 0.101) (Figure 3a). A *p*ost hoc pairwise test revealed that, after 30 min of rest, MDA levels tended to be lower in the S-LMWP phase compared to the placebo phase but did not reach statistical significance (*p* = 0.059) (Figure 3a). On the other hand, the levels of MDA were significantly higher in the 5-LMWP phase compared to S-LMWP (*p* = 0.022) (Figure 3a). In addition, the changed percentage of MDA levels was significantly lower in the S-LMWP phase compared to the placebo phase (*p* = 0.007) (Figure 3b).

### 3.8. Relationships Between Oxidative Stress-Related Markers and Biochemical Markers

**Figure 4:**
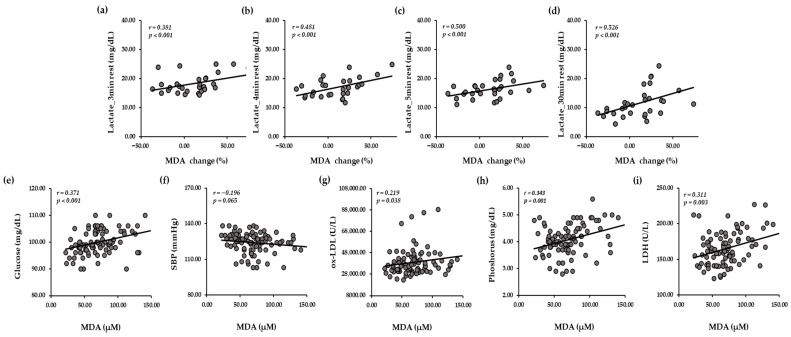
Relationships between oxidative stress-related markers and biochemical markers. Correlation between lactate levels at each time point and MDA change (%) (**a**–**d**), and correlation with pooling MDA levels and glucose (**e**), SBP (**f**), ox-LDL (**g**), phosphorus (**h**), and LDH levels (**i**). Correlation analyses were performed using Spearman correlation test (unadjusted). A *p*-value < 0.05 was considered statistically significant. MDA change (%) was the changed percentage of serum MDA levels before and at 30 min of rest after exercise. Rest, 30 min rest after the exercise; *r*, correlation coefficient; *p*, *p*-value; LDH, lactate dehydrogenase; MDA, malondialdehyde; ox-LDL, oxidized-LDL; SBP, systolic blood pressure.

Figure 4 shows the relationship between oxidative stress-related markers (MDA, ox-LDL) and biochemical markers (lactate, SBP, glucose, phosphorus, LDH) during the study period. Lactate levels were tested for correlation with the percentage of MDA changed before exercise and at 30 min of rest (Figure 4a–d), while SBP, glucose, ox-LDL, phosphorus, and LDH were analyzed for correlation with pooling MDA (Figure 4e–i). After exercise, significant positive relationships in lactate were observed with the changed percentage of serum MDA at 3, 4, 5, and 30 min of rest after exercise (*p* < 0.001). SBP levels were negatively but not significantly correlated with MDA levels (*p* = 0.065). In addition, serum levels of ox-LDL, glucose, phosphorus, and LDH were positively correlated with MDA levels (*p* < 0.05).

## 4. Discussion

This study aimed to investigate the effects of LMWP on fatigue and oxidative stress responses during a maximal exercise test in healthy men in their twenties. Recently, several studies have shown the antioxidant effects of LMWP [23,25,26,27,28,29,30,31,32]. However, there have been no studies on the changes in various biomarkers according to short-term consumption of LMWP following a maximal exercise test. To our knowledge, this is the first study to demonstrate that short-term LMWP consumption was associated with reduced fatigue and oxidative stress responses during a maximal exercise test.

It was reported that exercise-induced oxidative damage can negatively impact cellular membranes, leading to cellular swelling, reduced membrane fluidity, disruption of the ionic gradient, tissue inflammation, DNA damage, and protein modifications [36,37,38]. The elevated levels of MDA, a well-known marker of lipid peroxidation, have been associated with impaired muscle cell integrity and cellular dysfunction [36]. Thompson D et al. reported that, prior to a 90 min intermittent running test, subjects who consumed 400 mg of vitamin C for 2 weeks showed decreased MDA levels compared with the placebo group [39]. However, several studies showed that antioxidants were not effective components for exercise performance and muscle injury [40]. These conflicting results suggest the need for effective antioxidant compounds that can increase bioavailability, which is limited by differences in the level of absorption resulting from characteristics between individuals and species [41]. In the present study, increased MDA levels immediately after the exercise significantly returned to near-baseline levels at 30 min in the S-LMWP phase. These results suggest that a single intake of LMWP may help attenuate exercise-induced oxidative stress by promoting recovery of elevated MDA levels after exercise.

CK and LDH are commonly associated with muscle damage, and elevated serum concentrations are considered indicators of muscle cell membrane disruptions and damage to other tissue structures [42,43,44]. Howatson et al. reported no significant difference in CK levels between tart cherry juice and placebo groups until 48 h into recovery after a marathon; however, the CK response was correlated with the inflammatory response [45]. In the present study, the S-LMWP phase showed a greater increasing trend in CK levels compared with the placebo phase, although substantial inter-individual variability was observed. In addition, baseline CK levels before exercise were significantly higher in the S-LMWP phase than in the placebo phase. Previous studies have reported that exercise-induced CK levels returned to baseline more rapidly by supplementing with polyphenols for more than 7 days [46,47]. Therefore, the changes in CK levels observed during the S-LMWP phase may not be influenced by LMWP consumption, possibly due to the short supplementation period, which may have been insufficient to affect CK responses. Also, CK-MB, which has been suggested as a potential marker of type I muscle fiber damage [48], was measured in our study. Hooper DR et al. demonstrated that tart cherry extract decreased CK activity and CK-MB levels compared with the placebo group following intense resistance exercise [46]. CK-MB levels have also been reported to decrease following vitamin E consumption one hour before exercise [49]. In this study, CK-MB levels in both LMWP phases were lower than those in the placebo phase but did not reach statistical significance.

LDH is an enzyme that catalyzes the interconversion of lactate and pyruvate during anaerobic glucose metabolism in muscle contraction [50]. Under normal conditions, serum LDH activity reflects the metabolism of lactate and the extent of basal cell damage which is related with the muscle soreness after exercise [51]. The current study also confirmed that supplementation with LMWP for 5 days could significantly reduce LDH activity after a maximal exercise test. These results are partly supported by a previous study showing that consumption of oligomerized lychee fruit extract for 30 consecutive days significantly reduced exercise-induced increase in LDH activity [52].

Accumulation of lactate indicates the status in which production exceeds removal at maximal workloads, which also indicates an altered NAD^+^/NADH ratio and increased reliance on anaerobic glycolysis [53]. Such metabolic stress may contribute to increased oxidative stress by overwhelming antioxidant defense systems and promoting the accumulation of ROS [53]. Similarly, Davies et al. reported increases in free radicals and MDA levels in the muscle and liver of rats after exhaustive exercise [54]. Lovlin et al. reported a positive correlation between blood lactate concentration and plasma MDA (*r* = 0.51, *p* < 0.001) [53]. In our study, we also found a positive correlation between lactate levels and serum MDA levels at rest after exercise. These results show that serum MDA levels and lactate were significantly reduced at 30 min of rest in the S-LMWP phase compared to the placebo phase. Likewise, a previous study reported that the change (%) in blood lactate levels was significantly lower in the 7-day LMWP supplementation group and that there was improved power output during high-intensity intermittent exercise [32]. Interestingly, in this study, the 5-LMWP phase showed the highest HRmax and earlier attainment of AT, while blood lactate levels were significantly lower than those in the placebo after a 30 min rest. Therefore, these results suggest the 5-day consumption of LMWP support post-exercise recovery following a maximal exercise test.

Aerobic exercise can result in increased oxidative damage to tissues [55]. This damage can lead to the production of ox-LDL, which can promote endothelial inflammation and contribute to the development of arteriosclerosis [56]. In this study, ox-LDL levels increased at all phases following exercise. However, no significant differences related to LMWP intervention were observed after 30 min of recovery. Nevertheless, there was a significant recovery in MDA levels with short-term consumption of LMWP, suggesting that consumption of LMWP may help reduce oxidative stress induced by a maximal exercise test.

However, the present findings should be interpreted in the context of previous reports demonstrating limited or adverse effects of chronic antioxidant supplementation in exercise training. High-dose and long-term supplementation with vitamins C and E has been shown to attenuate exercise-induced ROS signaling, mitochondrial biogenesis, and endogenous antioxidant enzyme activation, thereby potentially impairing training adaptations [21]. These findings suggest that indiscriminate suppression of ROS may be counterproductive, particularly in high-performance athletes. In contrast, the present study focused on the acute and short-term consumption of low-molecular weight polyphenols, which are characterized by enhanced bioavailability and rapid systemic absorption. Importantly, LMWP supplementation did not alter exercise performance or maximal oxygen uptake, indicating that short-term intake did not interfere with physiological responses to maximal exercise. Instead, reductions in blood lactate and malondialdehyde levels during the recovery period suggest that LMWP may facilitate post-exercise metabolic recovery and attenuate excessive oxidative stress [17]. Notably, oxidative stress markers increased immediately after exercise in all conditions, but recovered toward baseline levels only in the LMWP phase, particularly following single-dose intake. This pattern supports the concept that LMWP may modulate, rather than abolish, exercise-induced oxidative stress responses [57]. Such modulation may be advantageous for recovery without disrupting redox-sensitive signaling pathways involved in exercise adaptation.

The maximal exercise test employed in this study was sufficient to induce marked metabolic and oxidative stress, as evidenced by increases in blood lactate and oxidative stress markers immediately after exercise. However, these responses remained within physiological ranges commonly observed during maximal or near-maximal exercise in healthy individuals and should not be interpreted as indicators of tissue damage or pathological stress [37]. It is well-established that elevations in lactate, ROS, and muscle damage-related enzymes during strenuous exercise represent normal physiological signals that contribute to fatigue perception and act as protective mechanisms to limit excessive exercise intensity [40]. Such responses are essential for maintaining homeostasis and preventing organ and tissue injury during sustained high-intensity activity. In this context, the observed effects of LMWP supplementation should not be interpreted as suppression of these beneficial physiological signals. LMWP supplementation may facilitate post-exercise metabolic recovery without altering the normal stress responses elicited during exercise [58].

There are a few limitations in this study. First, although the study employed a crossover design with statistical power calculation, the sample size was relatively small and limited to healthy young men. Therefore, the generalizability of the findings to other populations, such as women, older adults, or trained athletes is limited. Second, participants were instructed to maintain their habitual diet and lifestyle during the intervention period in order to preserve consistent experimental conditions; however, strict dietary control was not implemented. Therefore, inter-individual differences in habitual dietary patterns may have influenced the results. Future studies evaluating the effects of antioxidant compounds should more rigorously control individual dietary intake and supplement use. Third, we did not assess changes in lipid profiles following LMWP ingestion after maximal exercise testing. As noted previously, exercise induces oxidative stress, which can lead to oxidative damage and may influence circulating lipid levels [40]. Future research should investigate the effects of LMWP consumption on lipid profiles, including lipoprotein fractions. Fourth, although the exercise protocol was designed to induce maximal physiological stress, the measured metabolic and oxidative biomarkers remained within normal physiological ranges, and direct indicators of tissue damage were not evaluated. Therefore, the observed effects should be interpreted as alterations in recovery kinetics rather than the prevention of overt exercise-induced injury. Finally, the supplementation period was relatively short, limiting the ability to assess long-term adaptations or training effects. Additional studies are needed to evaluate the effects of prolonged supplementation.

## 5. Conclusions

This is the first study to report that short-term LMWP (oligonol) consumption was associated with lower blood lactate levels during the recovery period and more rapid normalization of oxidative stress markers, although it did not affect exercise performance or immediate physiological responses to maximal exercise. These findings suggest that short-term LMWP (oligonol) consumption does not directly enhance exercise performance or attenuate normal exercise-induced physiological stress responses, but may facilitate post-exercise recovery following maximal exercise in healthy men in their twenties. Future studies should include more diverse and larger populations to improve the generalizability of these findings. In addition, more stringent dietary control criteria will be necessary to ensure methodological rigor, and a broader range of parameters should be measured to further evaluate the effects of low-molecular weight polyphenols (LMWPs) in the context of exercise.

## Figures and Tables

**Table 1:** General characteristics and NQ of subjects at baseline (*n* = 10).

Variables	Mean	±	SE	Min	Max
Age (years)	24.00	±	0.52	22.00	27.00
Height (cm)	177.47	±	1.87	166.70	188.10
Weight (kg)	80.32	±	2.79	67.70	93.50
BMI (kg/m^2^)	25.52	±	0.81	23.50	30.00
LBM (kg)	61.34	±	1.93	50.90	70.30
SMM (kg)	34.39	±	1.10	28.50	39.80
BFM (kg)	18.98	±	1.89	8.90	28.80
BFP (%)	23.37	±	1.75	11.20	30.80
NQ1 (balance)	28.88	±	4.45	3.93	47.00
NQ2 (moderation)	38.76	±	3.17	25.90	53.90
NQ3 (practice)	58.82	±	2.41	48.06	67.76
NQ4 (total)	43.82	±	2.18	34.90	56.57

Data are presented as mean ± standard error, minimum (Min) and maximum (Max). BMI, body mass index; BFM, body fat mass; BFP, body fat percentage; LBM, lean body mass; NQ, nutrition quotient; SMM, skeletal muscle mass.

**Table 2:** Comparison of anthropometric parameters according to LMWP consumption (*n* = 10).

Variables	Placebo			S-LMWP			5-LMWP			*p-*Value
Weight (kg)	80.32	±	2.79 ^ab^	80.87	±	3.01 ^a^	80.35	±	2.98 ^b^	0.125
BMI (kg/m^2^)	25.52	±	0.81 ^ab^	25.80	±	0.85 ^a^	25.57	±	0.86 ^b^	0.124
LBM (kg)	61.34	±	1.93	62.23	±	2.01	61.66	±	1.89	0.452
SMM (kg)	34.39	±	1.10	34.91	±	1.14	34.59	±	1.07	0.452
BFM (kg)	18.98	±	1.89 ^a^	18.64	±	1.92 ^b^	18.69	±	2.00 ^ab^	0.358
BFP (%)	23.37	±	1.75 ^a^	22.78	±	1.71 ^b^	22.94	±	1.07 ^ab^	0.031

Data are presented as mean ± standard error. *p*-values < 0.05 represent overall differences tested by the Friedman test (non-parametric ANOVA) for overall comparison. Post hoc pairwise comparisons were performed using Wilcoxon signed-rank test (non-parametric paired *t*-test), and identical letters denote groups that are not significantly different according to the post hoc analysis (*p* < 0.05). All anthropometric variables were measured before the exercise load test. BMI, body mass index; BFM, body fat mass; BFP, body fat percentage; LBM, lean body mass; 5-LMWP, 5-day consumption of low-molecular weight polyphenol; S-LMWP, single consumption of low-molecular weight polyphenol; SMM, skeletal muscle mass.

**Table 3:** Comparison of exercise performance and glucose level, blood pressure according to LMWP consumption (*n* = 10).

Variables	Placebo			S-LMWP			5-LMWP			*p*-Value
VO_2_max (mL/kg/min)	47.29	±	1.39	47.85	±	1.63	47.06	±	1.41	0.273
HRmax (bpm)	190.80	±	2.21 ^ab^	189.30	±	1.92 ^b^	193.00	±	1.61 ^a^	0.006
ET (s)	736.00	±	12.49	741.50	±	18.80	755.50	±	16.37	0.085
AT (s)	502.50	±	19.99	508.00	±	23.43	486.50	±	21.20	0.132
GL (kg)	43.41	±	1.25 ^a^	41.21	±	1.32 ^b^	40.22	±	0.88 ^b^	0.003
GR (kg)	45.87	±	1.93 ^a^	43.77	±	2.23 ^b^	42.87	±	2.13 ^b^	0.061
BMS (kg)	152.45	±	5.94	143.60	±	6.26	146.00	±	8.21	0.122
Glucose (mg/dL)	98.40	±	1.07	99.80	±	1.82	101.60	±	1.31	0.256
SBP (mmHg)	129.10	±	1.57 ^a^	122.40	±	3.21 ^ab^	120.20	±	2.47 ^b^	0.014
DBP (mmHg)	80.50	±	3.46 ^a^	73.70	±	2.24 ^b^	73.40	±	1.78 ^b^	0.199

Data are presented as mean ± standard error. *p*-values < 0.05 represent overall differences tested by the Friedman test (non-parametric ANOVA) for overall comparison. Post hoc pairwise comparisons were performed using Wilcoxon signed-rank test (non-parametric paired *t*-test), and identical letters indicate groups that are not significantly different according to the post hoc analysis (*p* < 0.05). Heart rate and VO_2_max were measured during the exercise load test, while blood glucose, blood pressure, and muscle strength were measured after the exercise load test. AT, anaerobic threshold time; BMS, back muscle strength; DBP, diastolic blood pressure; ET, exercise time; GR, right-hand grip strength; GL, left-hand grip strength; HRmax, maximal heart rate; 5-LMWP, 5-day consumption of low-molecular weight polyphenol; S-LMWP, single consumption of low-molecular weight polyphenol; SBP, systolic blood pressure; VO_2_max, maximal oxygen consumption.

**Table 4:** Comparison of heart rate changes indicating exercise performance according to LMWP consumption (*n* = 10).

Time	Placebo			S-LMWP			5-LMWP			*p*0	*p*1
Exercise										<0.001	0.293
Before	65.60	±	2.57	62.70	±	1.78	63.40	±	1.63		
After	191.30	±	2.07	189.30	±	1.92	192.00	±	1.50		
Rest											
1 min	116.30	±	4.97 ^b^	123.70	±	1.97 ^ab^	130.80	±	3.97 ^a^		
2 min	111.90	±	4.57 ^b^	117.10	±	2.12 ^ab^	120.50	±	3.22 ^a^		
3 min	108.90	±	5.69	111.10	±	1.68	115.00	±	2.80		
4 min	113.40	±	3.31	110.20	±	2.16	115.70	±	2.53		
5 min	110.60	±	3.04 ^ab^	107.00	±	2.03 ^b^	113.50	±	1.95 ^a^		
30 min	96.50	±	3.84	96.10	±	3.47	102.10	±	2.61		

Data are presented as mean ± standard error. A *p*-value < 0.05 was considered statistically significant. The time effect (*p*0) and the time × treatment interaction effect (*p*1) were evaluated using repeated-measures ANOVA based on Wilks’s lambda. Post hoc pairwise comparisons were performed using Wilcoxon signed-rank test (non-parametric paired *t*-test), and identical letters denote groups that are not significantly different according to the post hoc analysis (*p* < 0.05). 5-LMWP, 5-day consumption of low-molecular weight polyphenol; S-LMWP, single consumption of low-molecular weight polyphenol.

**Table 5:** Comparison of fatigue metabolism and oxidative stress-related markers levels according to LMWP consumption (*n* = 10).

Variables		Placebo			S-LMWP			5-LMWP			*p*-Value	
Each timepoint value											*p*0	*p*1
UA (mg/dL)	Before	6.71	±	0.27	6.57	±	0.16	6.50	±	0.23	<0.001	0.089
	After	6.56	±	0.26	6.60	±	0.19	6.56	±	0.23		
	Rest	8.37	±	0.34 ^a^	7.85	±	0.31 ^b^	8.27	±	0.33 ^ab^		
CK (U/L)	Before	153.60	±	18.41 ^b^	203.00	±	32.40 ^a^	139.80	±	11.70 ^ab^	<0.001	0.008
	After	178.10	±	21.66 ^b^	238.40	±	39.26 ^a^	167.20	±	14.73 ^b^		
	Rest	163.20	±	19.53 ^b^	211.20	±	33.77 ^a^	152.40	±	12.73 ^ab^		
CK-MB (ng/dL)	Before	1.64	±	0.20	1.48	±	0.14	1.43	±	0.16	<0.001	0.139
	After	1.89	±	0.24	1.74	±	0.17	1.71	±	0.20		
	Rest	1.70	±	0.20	1.57	±	0.16	1.58	±	0.17		
ox-LDL (mU/L)	Before	35,985.37	±	2515.65	35,152.11	±	2672.22	34,060.01	±	3167.76	<0.001	0.250
	After	44,276.02	±	4940.21	42,444.66	±	2452.35	43,410.29	±	5461.12		
	Rest	40,597.81	±	4153.36	36,440.74	±	2478.22	40,847.47	±	4772.95		
Change values (Δ)											*p*2	
UA (mg/dL)		1.66	±	0.40	1.28	±	0.31	1.77	±	0.24	0.388	
CK (U/L)		9.60	±	1.33	8.20	±	2.66	12.60	±	1.85	0.273	
CK-MB (ng/dL)		0.06	±	0.03 ^b^	0.10	±	0.03 ^ab^	0.15	±	0.02 ^a^	0.041	
ox-LDL (mU/L)		4612.44	±	2686.06 ^ab^	1288.64	±	2140.35 ^b^	6787.46	±	2592.04 ^a^	0.006	

Data are presented as mean ± standard error. A *p*-value < 0.05 was considered statistically significant. The time effect (*p*0) and the time × treatment interaction effect (*p*1) were evaluated using repeated-measures ANOVA based on Wilks’s lambda. The overall comparison of change values among the three phases (*p*2) was performed using the Friedman test (non-parametric ANOVA). Post hoc pairwise comparisons were conducted using Wilcoxon signed-rank test (non-parametric paired *t*-test), and identical letters indicate groups that are not significantly different according to the post hoc analysis (*p* < 0.05). After, immediately after the maximal exercise test; Before, before the maximal exercise test; CK, creatine kinase; CK-MB, creatine kinase myocardial band; 5-LMWP, 5-day consumption of low-molecular weight polyphenol; ox-LDL, oxidized-LDL; Rest, 30 min rest after the exercise; S-LMWP, single consumption of low-molecular weight polyphenol; UA, uric acid.

## Data Availability

The raw data supporting the conclusions of this article will be made available by the authors on request.
